# Optical imaging for early detection of cervical cancer: state of the art and perspectives

**DOI:** 10.1117/1.JBO.28.8.080902

**Published:** 2023-08-09

**Authors:** Alisha Rahaman, Arpitha Anantharaju, Karthika Jeyachandran, Repala Manideep, Uttam M. Pal

**Affiliations:** a Savitribai Phule Pune University, Department of Microbiology, Pune, Maharashtra, India; b Jawaharlal Institute of Postgraduate Medical Education and Research, Department of Obstetrics and Gynaecology, Puducherry, India; c Indian Institute of Information Technology, Design and Manufacturing, Kancheepuram, Department of Electronics and Communication Engineering, Chennai, Tamil Nadu, India

**Keywords:** cervical cancer diagnosis, optical imaging, multimodal imaging, polarization-sensitive, white light imaging, Raman spectroscopy, fluorescence imaging, point-of-care

## Abstract

**Significance:**

Cervical cancer is one of the major causes of death in females worldwide. HPV infection is the key cause of uncontrolled cell growth leading to cervical cancer. About 90% of cervical cancer is preventable because of the slow progression of the disease, giving a window of about 10 years for the precancerous lesion to be recognized and treated.

**Aim:**

The present challenges for cervical cancer diagnosis are interobserver variation in clinicians’ interpretation of visual inspection with acetic acid/visual inspection with Lugol’s iodine, cost of cytology-based screening, and lack of skilled clinicians. The optical modalities can assist in qualitatively and quantitatively analyzing the tissue to differentiate between cancerous and surrounding normal tissues.

**Approach:**

This work is on the recent advances in optical techniques for cervical cancer diagnosis, which promise to overcome the above-listed challenges faced by present screening techniques.

**Results:**

The optical modalities provide substantial measurable information in addition to the conventional colposcopy and Pap smear test to clinically aid the diagnosis.

**Conclusions:**

Recent optical modalities on fluorescence, multispectral imaging, polarization-sensitive imaging, microendoscopy, Raman spectroscopy, especially with the portable design and assisted by artificial intelligence, have a significant scope in the diagnosis of premalignant cervical cancer in future.

## 1 Introduction

Cervical cancer is one of the leading causes of mortality in females worldwide.^1^^,^^2^ The number of incidences and mortality of cervical cancer is estimated to increase by 32% (from 604,127 to 797,712) and 41% (from 341,831 to 481,451) between 2020 and 2040, as reported by GLOBOCAN 2020 Report.^1^^,^^3^ Human papillomavirus (HPV) infection is the key cause of uncontrolled cell growth leading to cervical cancer. About 90% of cervical cancer is preventable because of the slow progression of the disease, giving a window of about 10 years for the precancerous lesion to be recognized and treated.^4^^,^^5^ The challenges for cervical cancer diagnosis are inter-observer variation in clinician’s interpretation, cost of cytology-based screening, and lack of skilled clinicians.^6^^,^^7^

**Fig. 1:**
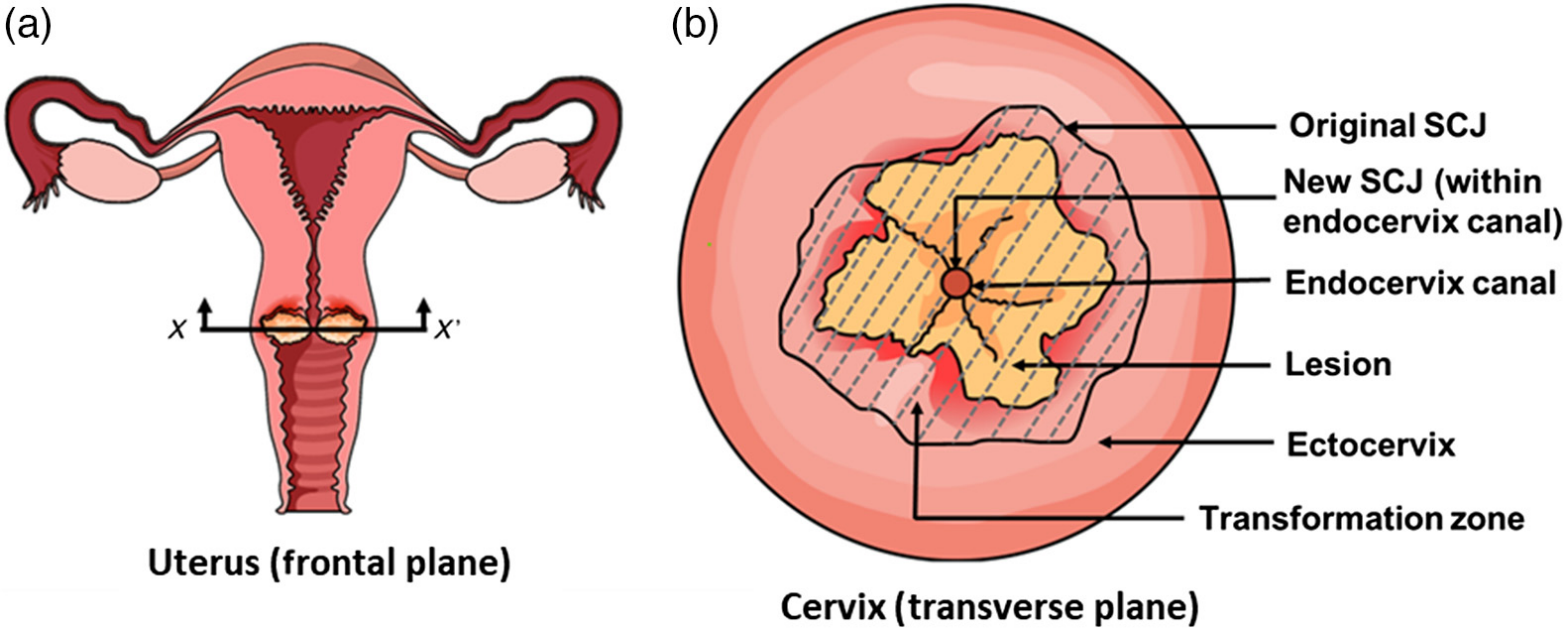
(a) Uterus (frontal plane) with diseased cervix (yellow), (b) diseased cervix (transverse plane) with the lesion in yellow and the transformation zone represented by the gray shaded area.

The cervix is divided into the endocervical canal and ectocervix, lined by granular and squamous epithelium, respectively. The junction between the two epitheliums is known as the squamocolumnar junction (SCJ). In a person, the SCJ is originally present in the ectocervix, which then moves toward the endocervical canal during adolescence and pregnancy, forming the new SCJ. The region of epithelium between the old and the newly formed SCJ is called the transformation zone (T-zone), which is also prone to develop cervical lesions, as shown in Figs. 1(a) and 1(b).^8^ According to World Health Organization guidelines, a general screening for cervical cancer in women should be started ideally at the age 30 years followed by a regular screening at every 5 to 10 years. In HIV-positive women, the screening should be done at the age of 25 years followed by regular screening at every 3 to 5 years.^5^

Conventional methods for detecting cervical cancer include direct methods, which are the gold standard techniques, such as the Pap-smear test and visual inspection with acetic acid and Lugol’s iodine.^2^^,^^9^^,^^10^ The Pap smear test involves the collection of cells from the transformation zone, which are stained and examined by clinicians to differentiate between normal and cancerous cells. The visual inspection, with or without colposcopy, involves assessing the cell’s nuclear morphology and reaction after applying acetic acid and Lugol’s iodine. The acetic acid applied on the surface of the cervical tissue denatures the protein and results in white patches, which can be observed visually. The growth, decay, and density of the white patch developed due to this coagulation of the proteins are used to delineate precancerous from normal. Lugol’s iodine quantifies the composition of the glycogen content, which is inversely proportional to the measure of cell division. The clinicians provide a Swede score based on five parameters: acetic acid uptake, margins, vessels, lesion size, and iodine uptake.^11^ If the Swede score exceeds five, the lesion is identified as a precancerous lesion requiring biopsy for pathological confirmation.

However, acetic acid is corrosive in nature and causes discomfort to patients on application. Moreover, the variation in the clinician’s interpretation yields false positive and false negative results, leading to lower positive predictive value.^12^^,^^13^ Assays such as polymerase chain reaction (PCR) and next-generation sequencing (NGS) detect the HPV viral genome, suggesting the presence or absence of HPV infection may or may not be diagnostic for cervical cancer as most of the infections are self-limiting and disappear with time, and a prolong persistence of viral infection alone may lead to cervical precancer and cancer. Various conventional indirect methods have been used to detect HPV strains by polymerase chain reaction–restriction fragment length polymorphism based assay,^14^ NGS assay,^15^ and antigen capture by lateral flow assay; however, these molecular diagnostic techniques are time-consuming and require tedious sample preparation.

**Fig. 2:**
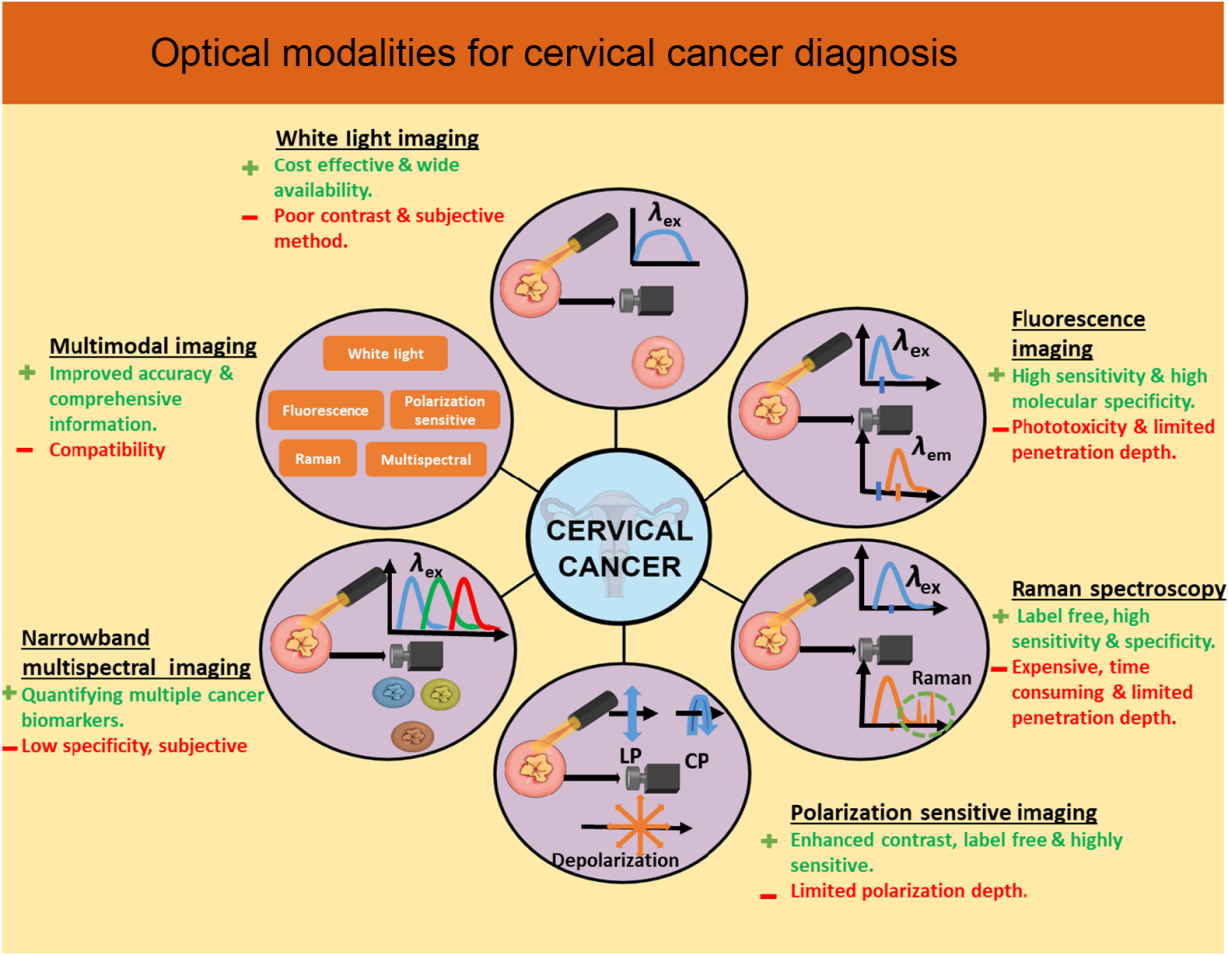
Schematic of various optical modalities for cervical cancer diagnosis.

Even though conventional diagnostic techniques have improved survival rates, there is a critical need for rapid and robust techniques that can be used in low-resource settings for accurate cervical cancer screening.^16^ Optical modality has been a key for cervical cancer diagnosis due to its cost-effectiveness, compactness, robustness, and reproducibility.^17^ Optical modalities have been used for breast cancer,^18^ skin cancer,^19^ oral cancer,^20^ gastrointestinal cancer,^21^ and bladder cancer.^22^ Several optical modalities have been implemented for cervical cancer diagnosis as well. Orfanoudaki et al. discussed the development of devices for better resolution images and the requirement for automated image analysis.^23^ Reviews carried out by Thekkek et al., Novikova et al, and Gonzalez et al. identified and described about the required development of portable, cost-effective optical devices for accurate diagnosis of the cervical cancer.^24^^–^^26^ This study highlights newer advancements in the development of probes and optical devices for point-of-care cervical cancer diagnosis at low-resource setting also combined with AI/ML for detailed image analysis. The key features of various optical techniques that have been developed for the detection of cervical precancer are highlighted, as shown in Fig. 2.

## 2 Optical Modalities for Cervical Cancer Detection

In recent years, there has been significant progress in optical modalities, such as multispectral imaging, fluorescence imaging, polarization sensitive imaging, and Raman spectroscopy, and clinical procedures, such as colposcopy and endoscopy, which leverage optical technologies. In this section, different optical modalities based on white light imaging and fluorescence imaging for the detection of cervical precancer are discussed, and summarized in Table 1.

**Table 1:** Summary of the optical modalities for cervical cancer diagnosis.

Modality	Reference	Excitation wavelength	Sample size	Sensitivity	Specificity	Comparative analysis
White light imaging	Asiedu et al.^27^^,^^28^	Broadband	200	81.3%	78.6%	• A user-friendly device for imaging and sample collection; data analysis using ML algorithms
						• The image capturing requires application of external agents, such as Lugol’s iodine and acetic acid
	Petersen et al.^29^	Broadband	824	NA	NA	• Supported visual inspection by EVA system that allowed real time monitoring and evaluation of cervical lesions
						• Critical image capturing is required and depends upon the use of external agents
	Acheampong et al.^30^	Broadband	90	NA	NA	• Detection of hr-HPV along with cervical lesions
	Nessa et al.^31^	Broadband	932	Gynocular: 52.8% to 61.1%	Gynocular: 52.9% to 65.6%	• The instrument can be used in low-resource settings and operated by least specialized skilled professional
				Colposcope: 52.8% to 61.1%	Colposcope: 53.4% to 66.16%	
Fluorescence imaging	Pierce et al.^32^	455 nm	174	97%	93%	• HRME study helped to eliminate false positive cases diagnosed by colposcopy
						• HRME was not able to prove its point-of-care nature for testing patients
	Schlosser et al.^33^	445 nm	62	NA	NA	• The instrument was able to differentiate between normal squamous epithelium and HSIL, and to discriminate among columnar, squamous, and stromal tissue. It also shows the quantitative analysis of captured images helps in better detection and differentiation of cervical precancer
						• Imaging is restricted to the upper layers of the cervix due to the restricted penetration depth of extrinsic fluorophores
	Sheikhzadeh et al.^34^	488 nm	47	92%- to 100%	66% to 92%	• Quantitative analysis of captured images helps in differentiation of normal tissue, low grade and high grade lesions
						• A possibility of false negative result due to random selection of cervix area during image acquisition
	Meena et al.^35^	405 nm	28	95%	95%	• Label free fluorescence technique
	Pouli et al.^36^	755 and 860 nm	25	93.3%	81.6% to 83.3%	• Label free fluorescence technique
						• In-depth analysis of structural and morphological changes associated with cancer development
						• *Ex-vivo* study on less samples, hence more detailed *in-vivo* study is required
	Ji et al.^37^	405 nm	71	90.9%	100%	• Label free fluorescence technique
						• Use of unsupervised ML method
	Wang et al.^38^	405 nm	24	NA	NA	• Label free fluorescence technique
						• In-depth analysis of metabolic changes associated with cancer, which helps in early diagnosis
Raman spectroscopy	Shaikh et al.^39^	785 nm	26	91%	96%	• Quantitative, non-invasive and highly sensitive
						• A long time is required to record spectrum from a large area
	Kanter et al.^40^	785 nm	90	92% to 98%	81% to 96%	• Raman spectrum along with the algorithm helps in identifying dysplasia
	Raja et al.^41^	785 nm	48	94.4% to 100%	96.7% to 100%	• Differentiation of normal and cancer patients based on the Raman spectrum of blood plasma
	Zhang et al.^42^	532 nm	93	NA	NA	• Rapid differentiation between cervical adenocarcinoma and cervical squamous cell carcinoma
						• Critical data analysis is required using various algorithms, which could have been combined and developed into a software
	Yang et al.^43^	532 nm	195	NA	NA	• Feature fusion combined with Raman spectroscopy helps in improving the classification between different grades of cervical cancer
Polarization-sensitive imaging	Roman et al.^44^	633 nm	22	NA	NA	• The low cost and rapid procedure of the instrument gives it an advantage to be used as a point-of-care device
	Kupinski et al.^45^	450 to 700 nm	24	NA	NA	• The study reports the high accuracy in the detection of cervical cancer.
	Zaffar et al.^46^	440 nm	28	NA	NA	• The polarization patterns helps in clear demarcation of different tissue types. This further helps to monitor the disease progression by observing the changing polarization pattern
	Zaffar et al.^47^	405 nm	60	90% to 100%	87% to 100%	• Along with a clear differentiation between normal and different grades of cervical cancer, the system also allows to monitor the disease progression
						• Invasive, requires cervical tissue sections
	Liu et al.^48^	532 nm	NA	NA	NA	• The method provides detailed features of nucleus and hence, would help in advancement of Pap smear test
	Roa et al.^49^	800 nm	16	NA	NA	• The system can differentiate between collagen and elastic fibers, which can help further in studying not only cervical cancer but the reproductive organs
						• Requires image pre-processing and further critical image analysis
NBI	Yi et al.^50^	415, 450, 525, and 620 nm	24	NA	NA	• Rapid capturing of multispectral images, simple algorithm for classification of normal and cancerous tissues
						• Invasive and requires clinician inputs to mark the lesions
	Kobara et al.^51^	415 and 540 nm	95	87%	50%	• Non-invasive, less discomfort to patients and performance similar to colposcopy
						• Low specificity as compared to colposcopy and performance is similar to that of colposcopy when detecting CIN2+ lesions
	Uchita et al.^52^	540 nm	24	100%	100%	• Non-invasive, differentiation between different grades of cervical intraepithelial neoplasms
Multimodal imaging	Coole et al.^53^	460 nm	NA	NA	NA	• Non-invasive, does not require biopsy, can be employed at point-of-care
	Gordon et al.^54^	Broadband	18	72.34%	67.62%	• Improve detection performance, ease of use

EVA, enhanced visual assessment; HRME, high resolution microendoscopy; HPV, human papilloma virus; CIN, cervical intra-epithelial neoplasia; HSIL, high grade squamous intraepithelial lesion.

### 2.1 White Light Imaging

White light imaging has been widely used to study cell and nuclear morphology. This modality provides a platform to visualize and study micro-sized cells and tissues to determine the underlying disease that affects cellular morphology and physiology. The visual inspection involves collecting the tissue biopsy, which is sliced in order of hundreds of micrometers and stained appropriately to assess the cancer biomarkers qualitatively.

**Fig. 3:**
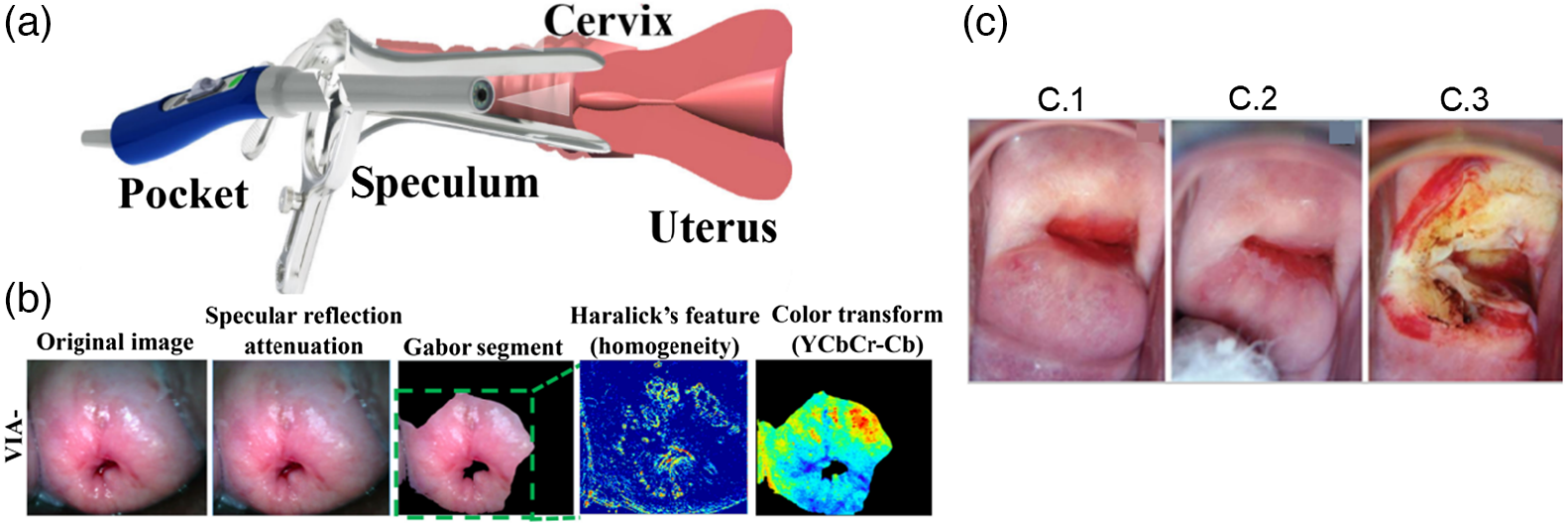
(a) Schematic of cervix imaging using a Pocket colposcope.^28^ (b) Steps of image analysis of cervical lesions with the help of visual inspection by acetic acid and Lugol’s iodine.^28^ (Reprinted from Ref. 28 with permission of IEEE © 2018). (b) Cervix image of a patient who tested positive for HPV: (c.1) before acetic acid, (c.2) after acetic acid, (c.3) treatment-thermal coagulation.^30^ (Reprinted from Ref. 30 with permission of *e*cancer © 2021.)

White light imaging techniques (visual inspection with acetic acid and visual inspection with Lugol’s iodine) also support the visual inspection with or without a colposcope. Here, we evaluate the degree of white patch intensity (due to acetic acid uptake) or yellow color intensity (due to Lugol’s iodine non-uptake) for initial cervical cancer screening. The recent development of white light imaging has shown the development of various types of transvaginal imaging probes for better visual inspection of the cervix. Asiedu et al. designed a user-friendly POCkeT colposcope that enables self-insertion and clinician-assisted image capturing of the cervix for cancer screening. The tool also enables easy application of acetic acid and Lugol’s iodine for further review.^27^ Aseidu et al. followed POCKeT with the developed machine learning (ML) algorithm that utilizes the images of acetic acid and Lugol’s iodine to treat the cervical tissues. The ML algorithm includes the selection of the region of interest of the cervix, reduction of specular reflectance, segmentation of selection region by Gabor algorithm, Haralick’s method for textural feature extraction, image processing based on different colors, and determination of the lesion size. Figures 3(a) and 3(c) show the image of the Pocket colposcope and the images captured by the system.^28^ Peterson et al. showed the application of a mobile-based colposcope with an enhanced visual assessment (EVA) system to support visual inspection by white light imaging for cancer screening.^29^ Acheampong et al. used a colposcope employed with an EVA system to detect cervical precancer in women while also assessing the prevalence of hr-HPV (high-risk human papillomavirus). The *in-vivo* images reported in the system are shown in Fig. 3(b), while the image processing steps are showcased in Fig. 3(c).^30^ A warm white LED-based white light source in a gynocular and colposcope device in a study performed by Nessa et al. for better detection of cervical lesions. The data collected by the devices were studied and analyzed by doctors to acquire information about acetowhiteness, lesion surface and size, glucose content, etc., to predict CIN 2+ (CIN 2, CIN 3, and invasive cervical cancer).^31^

### 2.2 Fluorescence Imaging

The fluorescence imaging modality consists of target-specific binding fluorophores and is used for obtaining qualitative and quantitative information about the desired target. The fluorescence imaging modality has higher sensitivity and resolution to detect cancer but is limited due to the lower intensity of fluorescence emitted by the fluorophores.^55^ The fluorescence imaging can be used as a label-free with endogenous fluorophores (NADH, flavins, porphyrins, and collagen) and label based with an exogenous fluorescent dye (ICG, fluorescein, rhodamine, and cyanine).

**Fig. 4:**
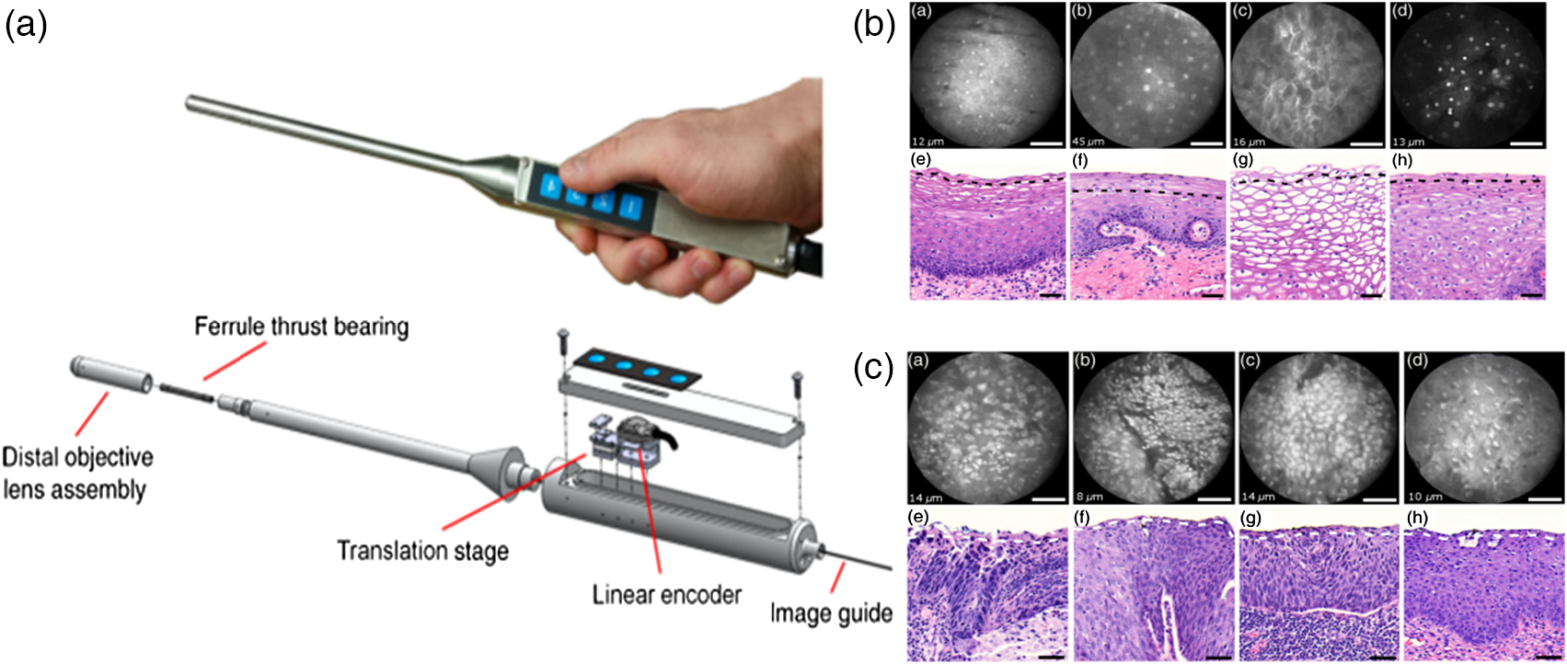
*In-vivo* fluorescence endomicroscopy. (a) Image of the fluorescence-based endomicroscope. (b) Endomicroscopic images of normal squamous epithelium compared with the histological images. (c) Endomicroscopic images of precancerous squamous epithelium compared with histological images.^33^ (Reprinted from Ref. 33 with permission of SPIE © 2016.)

**Fig. 5:**
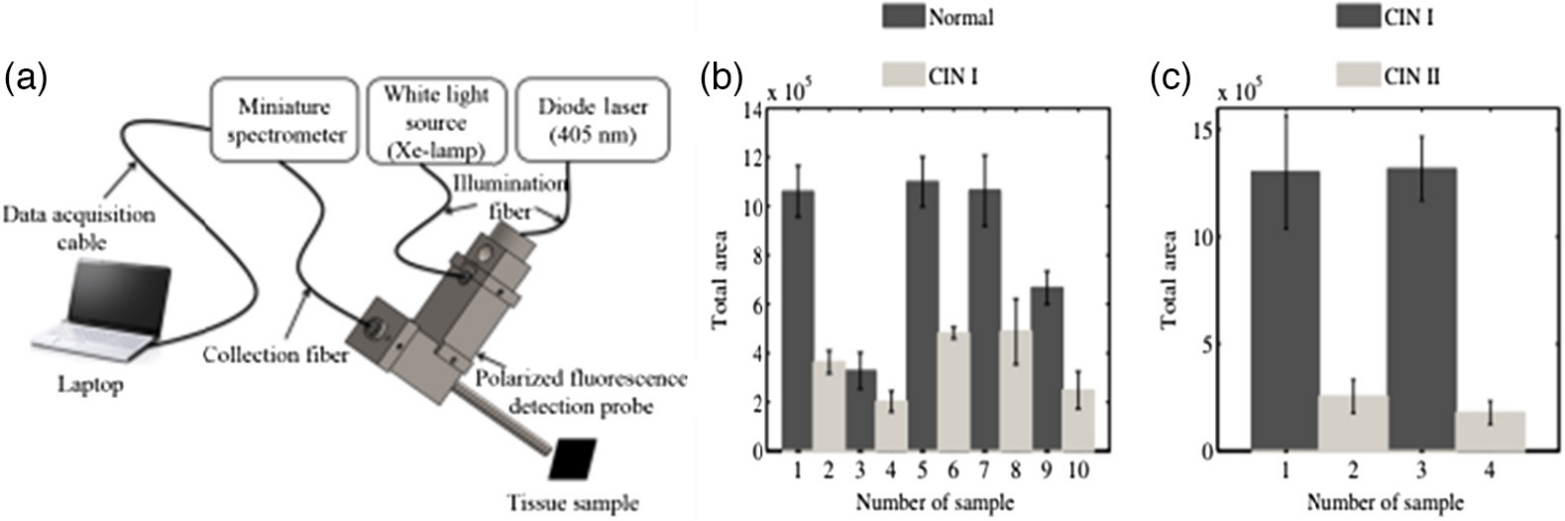
(a)–(c) Intrinsic fluorescence-based portable device for cervical cancer detection. (a) Design of the instrument, (b) comparison of intrinsic fluorescence intensity of normal and CIN-1 lesions obtained from whole uterus samples, and (c) comparison of intrinsic fluorescence intensity of CIN1 and CIN2 lesions.^35^ (Reprinted from Ref. 35 with permission of SPIE © 2018.)

Pierce et al. incorporated fluorescence imaging with a low-cost, high-resolution microendoscope (HRME). This technique required the use of an exogenous fluorescent dye, proflavine, that selectively stains cell nuclei and has an excitation and emission wavelength at 445 and 515 nm, respectively. HRME could identify the abnormality in the cervix by measuring the nuclear-cytoplasmic data *in vivo* and validating by biopsy studies of the same lesions.^32^ The capabilities of a fluorescence-based endomicroscopy to differentiate between normal columnar epithelium, stromal tissues, normal and precancerous squamous epithelium *in vivo* was explored and studied by Schlosser et al. In this study, acriflavine dye having an excitation wavelength of 445 nm was topically administered into the cervical region to measure the fluorescence emitted by the stained nuclei of the cells. The *in-vivo* images were then compared with the *ex-vivo* images of the biopsied cervical regions, which were then analyzed by stack analysis, as shown in Fig. 4(b). The authors reported that the device shown in Fig. 4(a) could assist in identifying subtle differences between normal and cancerous tissue types but cannot obtain and analyze data from deep tissues due to the lack of depth penetration of extrinsic fluorophores.^33^ Though fluorescence confocal microscopy cannot independently detect cervical cancer *in vivo*, promising results were shown in the diagnosis and screening of cervical cancer in *ex-vivo* conditions in a study by Sheikhzadeh et al. The diagnosis through the confocal images was validated using the histological images’ clinical interpretation. The instrument could obtain images at different depths of the cervical epithelium, such as below 30  μm and analyze the images with a 92% to 100% sensitivity. This indicates that the fluorescence confocal microscopy can differentiate between normal cervical tissue, low-grade cervical intraepithelial neoplasia (CIN), and high-grade CIN in *ex-vivo* biopsy tissue based on features, such as nuclear area, cell density, estimated nuclear-to-cytoplasmic (ENC) ratio, and the average distance between a nucleus and its three nearest Delaunay neighbors.^34^ A study by Meena et al. detects and differentiates between normal and cancerous samples *ex-vivo* by measuring the fluorescence of FAD and elastic scattering of whole uterus samples using the system shown in Fig. 5(a). PCA and LDA analyzed the measured fluorescence and elastic scattering to differentiate between normal, CIN1, and CIN2. Figure 5(b) shows the difference in intrinsic fluorescence in normal, CIN1, and CIN2 lesions. This study raises significant interest in developing a similar probe for *in-vivo* detection and diagnosis of cervical cancer.^35^

Studies have been carried out to overcome the use of external fluorophores, also known as label-free fluorescence lifetime imaging (FLIM). The technique takes advantage of the autofluorescence property of NAD(P)H and FAD to obtain high-resolution images of the cervix region. Pouli et al. used label-free two-photon excited fluorescence technique to image the freshly excised biopsy samples for imaging and employed a laser of excitation wavelength 755 and 860 nm, and the emission is detected at 460±20 and 525±25  nm for NAD(P)H and FAD, respectively. The images are analyzed to observe the epithelial thickness, mitochondrial organization, and changes in the cellular metabolic changes as a marker to differentiate and classify between normal, low grade, and high grade squamous intraepithelial lesions (SIL).^36^ Ji et al. combined FLIM with ML algorithms for imaging of exfoliated cervical cell samples that helps in predicting risk of cervical cancer in the patients with a sensitivity and specificity of 90.9% and 100%, respectively.^37^ Wang et al. used phasor analysis for the image analysis obtained from FLIM that helps in distinguishing normal, low, and, high grade cervical lesions based on the relationship between metabolic changes and cancer development.^38^ Qiu et al. performed a strong near-infrared absorption based fluorescence imaging for the early diagnosis of cervical cancer.^56^

### 2.3 Raman Spectroscopy

**Fig. 6:**
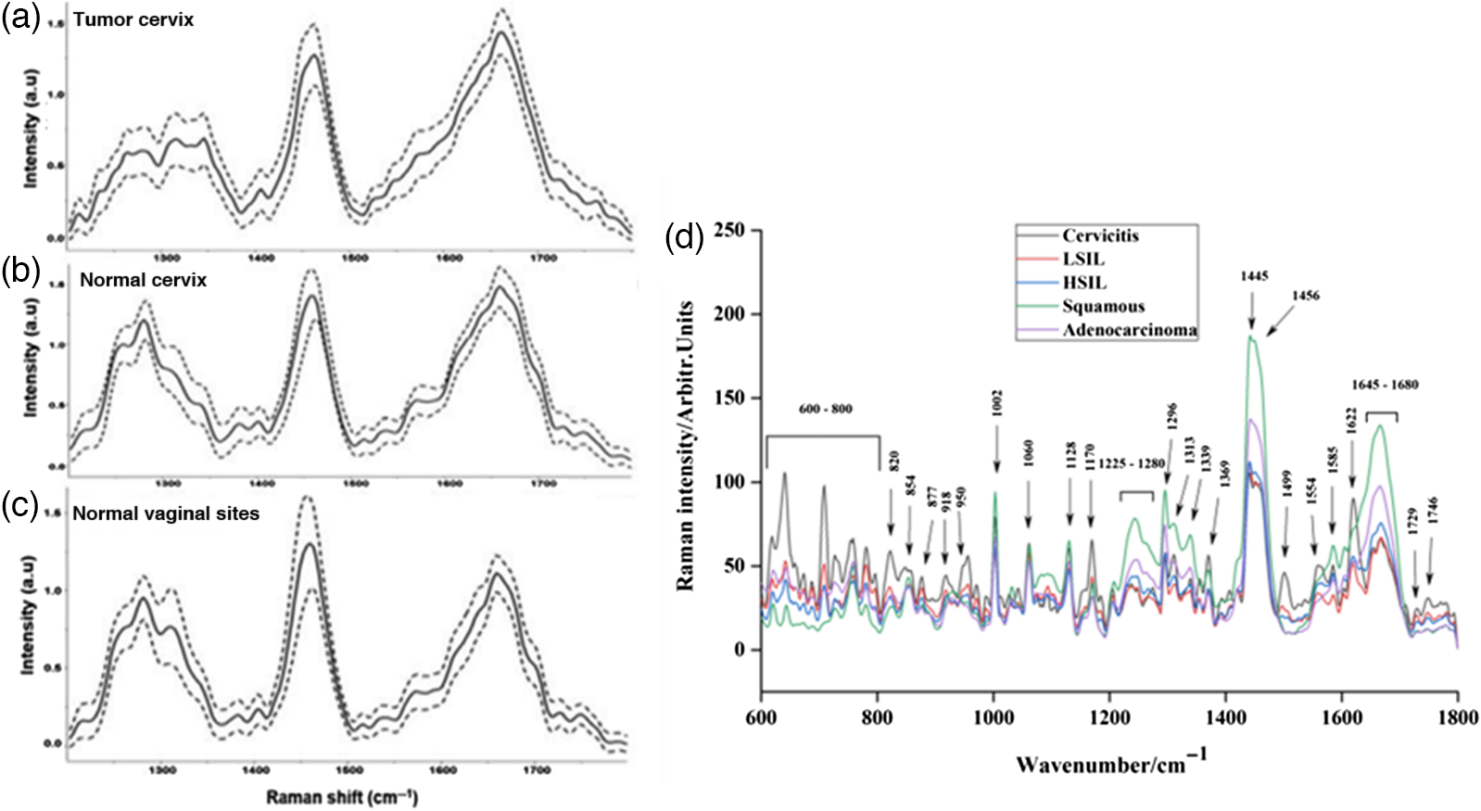
(a) Cancerous site of cervix, (b) normal cervical tissues, and (c) normal vaginal sites.^39^ (Reprinted from Ref. 39 with permission of Wiley © 2017.) (d) Spectral differences between cervicitis, LSIL, HSIL, cervical squamous cell carcinoma, and cervical adenocarcinoma.^43^ (Reprinted from Ref. 43 with permission of Wiley © 2021.) (LSIL, low-grade squamous intraepithelial lesion; HSIL, high-grade squamous intraepithelial lesion.)

Raman spectroscopy has been extensively explored for the early detection of cancers. In the study by Shaikh et al., the authors studied the ability of Raman spectroscopy to detect cervical cancer. The study revealed that the Raman spectroscopy was a better method for non-invasive cancer at low-resource settings by identifying peak intensity differences for hemoglobin and non-collagenous proteins between tumorous cervical tissues and normal tissues, as shown in Figs. 6(a)–6(c).^39^ Raman spectroscopy for *in-vivo* detection of cervical cancer based on peak differences at 1006, 1058, 1086, 1244, 1270, 1324, 1450, 1550, and $1655\;{\mathrm{cm}}^{-1}$ has been used to classify high-grade dysplasia, low-grade dysplasia, squamous metaplasia, and normal ectocervix using multiclass diagnostic algorithms, respectively.^40^ Cervical cancer has also been differentiated from normal samples by using near-infrared (NIR) based Raman spectroscopy in the study performed by Raja et al. In this study, blood samples of patients diagnosed with cervical cancer and normal patients were used to collect Raman signals. The signals from the normal and cancerous blood samples were compared, indicating that the peak intensity of some amino acids, such as phenylalanine, tryptophan, and tyrosine, and nucleic acids, such as adenine, increases in the case of cancer, a basis for considering as cancer biomarkers.^41^ Zhang et al., in their study, took formalin-fixed cervical tissue samples collected from patients diagnosed with cervical adenocarcinoma and cervical squamous cell carcinoma. These samples were used to collect the Raman spectrum, which was compared and analyzed using multiple ML algorithms, such as adaptive iteratively reweighted penalized least squares (airPLS) algorithm, Vancouver Raman algorithm, partial least squares (PLS), principal component analysis (PCA), kernel PCA, isometric feature mapping (isomap), and locally linear embedding. The algorithms were used to identify quantitative and qualitative differences between the two Raman spectrums and classify the samples into the two types of cancers. Out of all the algorithms, airPLS-PLS-KNN shows the highest accuracy of 96% for screening cancer.^42^ A recent study by Yang et al. uses Raman spectroscopy to identify and differentiate various cervical tissues and related diseases. In the study, the authors developed a confocal Raman spectrometer with a laser excitation of 532 nm to record the spectrum of cervical tissue samples collected from patients suffering from cervicitis, low-grade and high-grade SIL, cervical squamous cell carcinoma, and cervical adenocarcinoma. The conditions mentioned above were differentiated based on the spectral differences of proteins, carbohydrates, and nucleic acids, as shown in Fig. 6(d), which was further analyzed and differentiated using linear and non-linear classification models.^43^

### 2.4 Polarization-Sensitive Spectroscopy

**Fig. 7:**
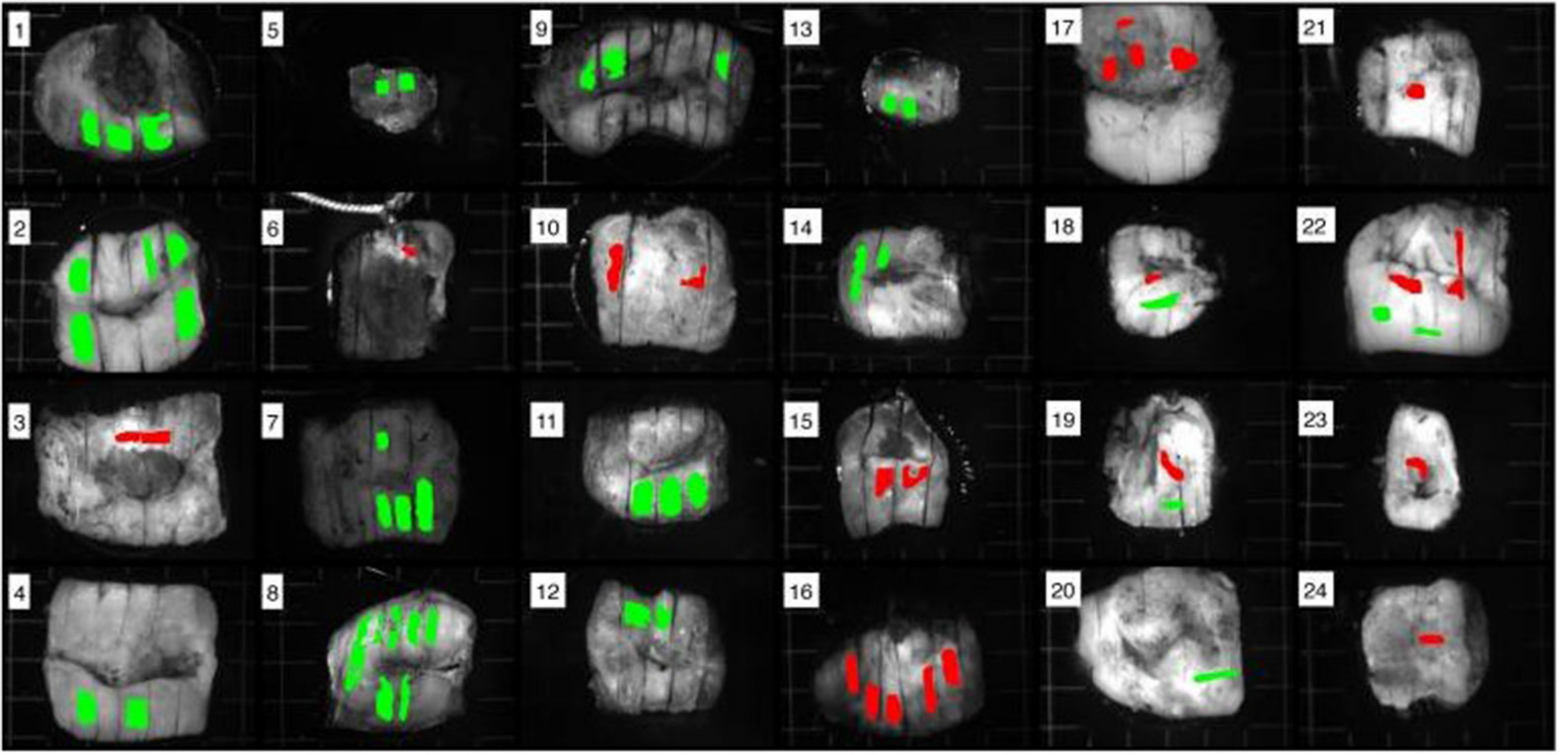
Radiance images (550 nm) of 24 patients labeled in red and green to represent high-grade CIN and healthy tissues, respectively, in accordance with histopathological studies.^46^ (Reprinted from Ref. 46 with permission of SPIE © 2018.)

Mueller matrix polarimeters have been recently studied for their ability to accurately diagnose cancer. The imaging system is highly sensitive and accurate, has high measurement speed, and can perform multicomponent analysis.^44^ Ramella-Roman et al. developed a snapshot Mueller matrix polarimeter that measures the polarization of collagen fibers in the cervix with the help of an LED operating at 633 nm was developed. The study reveals that any deviation from the standard polarization of collagen can indicate a diseased condition.^45^ Kupinski et al. developed a polarization-sensitive imaging technique while operating at 450 to 700 nm and acquiring 16 images at each wavelength to reconstruct the Mueller matrix of the sample. Radiance images acquired at 550 nm showed a low signal-to-noise ratio with high sensitivity and specificity. Figure 7 shows the images of 24 excised cervical samples in accordance with histopathological studies.^46^ Zaffar et al. describe that polarization dynamics can be studied to differentiate between epithelium and stroma of cervical tissue. The study further explains the differentiation between the two types of tissue based on the picosecond result intensity patterns at different time delays recorded with the Mueller matrix polarimeter.^47^ Zaffar et al., in the follow-up study, described that the orientations of collagen fibers randomize during disease progression. The orientations have been acquired in the form of matrix images, which have been analyzed using the Fourier domain. Based on the first row and column elements, the Fourier spectrum differentiates between cervical neoplasia (CIN-1, CIN-2) from the normal tissues.^48^ Liu et al. described the use of a polarization-based multi-parametric imaging system compared with conventional microscopy’s ability to resolve fine structural features of the nucleus in the cervical cells. This study sheds light on the application of polarization multi-parametric imaging to diagnose cervical precancers based on Pap-smear test and nuclear-cytoplasmic density.^49^ Roa et al. described the identification and classification of collagen and elastin fibers in cervical tissues based on the microscopic images acquired by Mueller matrix polarimeter combined with convolutional neural networks and K-nearest neighbor for classification. The study was performed on mice to observe and classify the different tissue fibers.^57^ Such studies prove the application of polarization-sensitive imaging to detect and diagnose cervical cancer based on different structural features, such as the orientation of the fibers and resulting anisotropy.

### 2.5 Narrow-Band Imaging

**Fig. 8:**
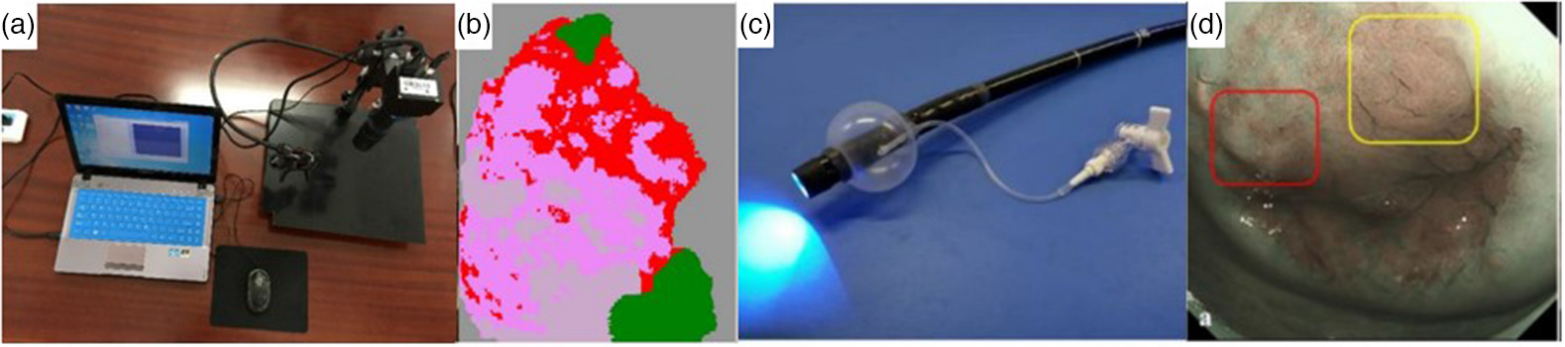
(a) Computer-aided narrow-band snapshot MSI system, (b) fused image of NBI snapshot processed with Euclidean classification algorithm in which green, gray, pink, and red regions indicate normal, CIN2, CIN3, carcinoma *in situ*, respectively.^50^ (Reprinted from Ref. 50 with permission of Springer © 2020.) (c) ME-NBI system, (d) NBI imaging showing thin white epithelium (yellow box) and CIN1 confirmed by biopsy (red box).^51^ (Reprinted from Ref. 51 with permission of MDPI © 2021.)

Narrow band imaging enables clear and high-resolution visualization of tissue structures, allowing a precise and enhanced diagnosis of lesions. The earliest work on the use of multispectral imaging for cervical cancer diagnosis was performed by Benavides et al. while identifying the critical operating wavelength range from 330 and 360 nm and 440 to 470 nm.^58^ Yi et al. developed a computer-aided multispectral narrow-band imaging (NBI) system, as shown in Fig. 8(a), operating at four wavelengths 415, 450, 525, and 620 nm to capture multiple images of the cervix at zero-lag time. The images were then fused and processed using the Euclidean distance classification algorithm to classify normal and cancerous tissues, as shown in Fig. 8(b). The main advantage of this technique is that it does not require external reagents to stain the cervix and diagnose cancer.^50^ Kobara et al. compared NBI with colposcopy in detecting CIN and CIN2+ lesions. The study showed that NBI is highly sensitive (87%) than colposcopy (74%) but has a low specificity (50%) when compared with that of colposcopy (68%). It also describes that NBI performs similarly to colposcopy while detecting CIN2+ lesions. Figure 8(c) shows the system’s image, and Fig. 8(d) shows the difference between a colposcopic image and NBI image.^51^ Uchita et al. described cervical cancer screening by combining the magnifying endoscopy and narrow-band imaging (ME-NBI). The differentiation between CIN and non-CIN tissues was made based on the presence of light white epithelium and heavy white epithelium with atypical intra-epithelial papillary capillary loop. The ME-NBI system enabled the clear visualization of tissue structures.^52^

### 2.6 Multimodal Techniques

**Fig. 9:**
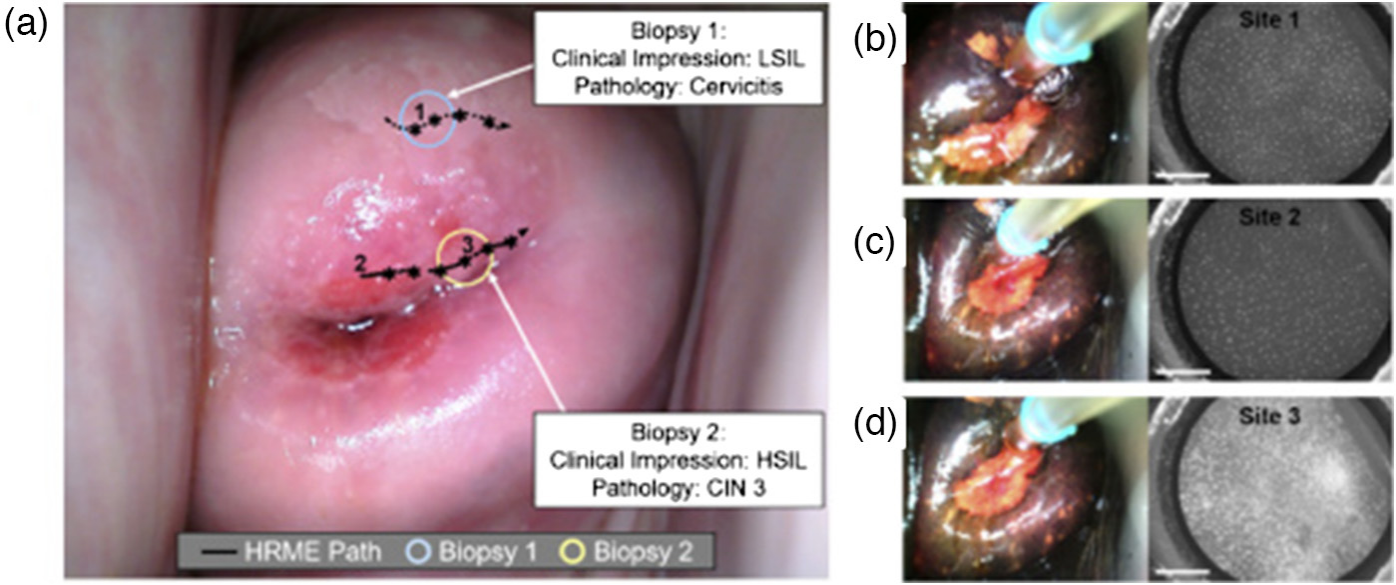
(a) MMC image of cervix of patient with low grade and high grade cervical lesions showing acetowhiteness. (b) Images of low grade lesion. (c) Images of normal cervical region. (d) Images of high grade lesion.^53^ (Reprinted from Ref. 53 with permission of OPTICA © 2022.)

Although single-modal imaging systems and detection probes provide significant information to the clinician for the diagnosis of cervical cancer, the multimodal approach helps in further validation and confirmation of the data obtained for higher accuracy in diagnosing cervical cancer. Coole et al. reported a multimodal imaging system for detecting cervical precancerous lesions. The system consists of a POCket colposcope integrated with HRME, which brings together the ability to measure the acetowhiteness of the lesions and fluorescence from the nuclei. The system employed an illuminator of a wavelength of 460 nm to excite the fluoropore proflavine dye that stains the nuclei. The images obtained, as shown in Figs. 9(a)–9(d), are analyzed by multi-task neural network that studied the nuclear characteristics and classified high-grade and low-grade cervical precancer or cancer.^53^ Gordon et al. detected SIL in the cervix region at an early stage by combining three different modalities into a probe system. The Micro Colposcope Probe detects SIL by combining white light reflected spectroscopy, coherent light scattering, and high-magnification, high-resolution RGB imaging. The probe consisted of four LEDs, a monochromatic infrared laser, and an RGB camera. Positive samples differentiated the negative samples by observing flat spectral signals, dark, and less textured scattering images, and patterns of blood vessels.^54^

## 3 AI Approaches for Cervical Cancer diagnosis

Artificial intelligence (AI) and ML techniques have shown great potential in the field of medical image analysis, including the detection and diagnosis of cervical cancer. Recent advances in AI/ML algorithms have led to significant improvements in accuracy, speed, and efficiency in cervical cancer diagnosis. AI/ML algorithms can analyze colposcopy images and provide an accurate diagnosis, reducing the need for unnecessary biopsies.

Jayabk et al. utilized image registration, feature extraction, and morphological operations to identify cancerous and non-cancerous cervical images with an accuracy of 97.14% and 100%, respectively. The adaptive neuro-fuzzy inference system was used as the method for classification.^59^ Kim et al. used filtering, preprocessing, and normalization techniques to extract AI features for cervical cancer detection. The authors developed the Cerviray AI and achieved an overall accuracy of 72% with a specificity of 83.05%.^60^ Wong et al. used hr-HPV testing, testing and training data, and classification methods, such as decision tree, random forest (RF), and SVM to classify CIN1, CIN2, CIN3, and CIN3+ with an accuracy of 96.88% and specificity of 94.32%.^61^ Tian et al. focused on the identification of HPV genotypes and somatic mutations for the diagnosis of cervical cancer. The authors utilized an RF unsupervised cluster to identify 17 types of HPV with an accuracy of 81.4%.^62^ Mehmood et al. applied data cleaning and feature selection techniques using the RF algorithm, forward propagation, backward propagation, and shallow neural network to diagnose cervical cancer. The algorithm used the Pearson correlation method to identify the most significant features for classification.^63^

Overall, these studies demonstrate the potential of AI/ML in the accurate and timely diagnosis of cervical cancer. The various techniques and methods employed in these studies can be combined and expanded to develop more robust and effective cervical cancer diagnosis tools. These approaches have been tabulated in Table 2.

**Table 2:** Summary of various ML approaches toward cervical cancer diagnosis.

References	Features	Methods	Classes	N	Accuracy	Sensitivity	Specificity
Jayabk et al.^59^	Image registration, feature extraction, morphological operations	Adaptive neuro-fuzzy inference systems	Cancerous cervical and non-cancerous cervical image	50	Non-cancerous: 97.14%	97.42%	99.36%
					Cancerous: 100%		
Kim et al.^60^	Filtering, preprocessing, and normalization, AI feature extraction, cervical cancer detection	The Cerviray AI	Normal, CIN1, CIN2, CIN3, cancer	10,000	72%	74.14%	83.05%
G.W.Wong et al.^61^	Hr-HPV test, testing and training data 80:20, classification	Decision tree, RF SVM-linear SVM-nonlinear	CIN1-, CIN2, CIN3, CIN3+	605	96.88%	90.91%	94.32%
Tian et al.^62^	Bio-specimen collection and DNA extraction, capture hybridization and NGS data generation, HPV genotypes detection and integration analysis, Genome Analysis Toolkit pipeline for somatic mutation identification	RF unsupervised clustering	17 types of HPV	34	81.14%	N/A	N/A
Mehmood et al.^63^	Data cleaning feature selection through RF, forward propagation, backward propagation, RF shallow neural network, diagnosis	The RF Pearson correlation	Cancerous and non-cancerous	858	93.6%	N/A	N/A

## 4 Conclusion

The studies described in this article individually have significant potential to be used as a screening technique for cervical cancer. However, there are also disadvantages, such as invasiveness, reproducibility of imaging system, interpatient variation, time-consuming sample preparation, variation in clinician’s interpretation, trained clinicians, and data scientists for image processing and diagnosis. While the analysis can be made simpler using AI where the ML algorithms could process the images and predict the disease as a normal, low-grade lesion or high-grade lesion with high accuracy. In such scenario, a cervical screening probe can be used as a point-of-care testing device.

Raman spectroscopy, combined with other techniques, serves as another alternative to multimodal imaging systems due to its ability to detect any tissue changes based on the spectra. Data analysis for Raman spectroscopy can be better supported with ML algorithms and databases. Apart from these, multimodal systems overcome the disadvantages and limitations of the individual systems by each system supporting the other system. The multimodal system combining the individual sensitivity of two or more techniques gives an accumulated higher accuracy to differentiate between a normal tissue and a precancerous lesion and a confirmed diagnosis of the disease, thereby reducing false positives and negative results. The AI/ML techniques have shown great potential in improving the accuracy and efficiency of a cervical cancer diagnosis. However, further research is required to validate these techniques in large-scale clinical trials and integrate them into routine clinical practice. With continued research and development, AI/ML along with multimodal optical approaches can significantly reduce the burden of cervical cancer worldwide.
